# Supplementary material for: De novo genome assembly of Bacillus altitudinis 19RS3 and Bacillus altitudinis T5S-T4, two plant growth-promoting bacteria isolated from Ilex paraguariensis St. Hil. (yerba mate)
Source: PLoS One. 2021 Mar 11;16(3):e0248274. doi: 10.1371/journal.pone.0248274 (PMC7954119; doi:10.1371/journal.pone.0248274)
Supplement: S1 Table — (DOCX) [file pone.0248274.s001.docx]

| **S1 Table.** Assembled genome quality statistics obtained for *Bacillus altitudinis* 19RS3 a plant growth-promoting bacterium isolated from *Ilex paraguariensis* St. Hil. using ABySS assembler. | | | | | | | | | | | | | | | | |
| --- | --- | --- | --- | --- | --- | --- | --- | --- | --- | --- | --- | --- | --- | --- | --- | --- |
| Statistics | k-mer 63 | k-mer 64 | k-mer 65 | k-mer 67 | k-mer 69 | k-mer 71 | k-mer 73 | k-mer 75 | k-mer 77 | k-mer 79 | k-mer 81 | k-mer 83 | k-mer 85 | k-mer 87 | k-mer 89 | k-mer 91 |
| # contigs (>= 0 bp) | 188 | 178 | 179 | 168 | 167 | 169 | 166 | 155 | 132 | 123 | 119 | 107 | 93 | 93 | 89 | 84 |
| # contigs (>= 1000 bp) | 16 | 16 | 17 | 17 | 17 | 17 | 17 | 15 | 14 | 15 | 15 | 13 | 14 | 14 | 15 | 15 |
| Total length (>= 0 bp) | 3821084 | 3820357 | 3820922 | 3821881 | 3822320 | 3822994 | 3823217 | 3821727 | 3820456 | 3819184 | 3822160 | 3822998 | 3824432 | 3821236 | 3823996 | 3823632 |
| Total length (>= 1000 bp) | 3793988 | 3794010 | 3793978 | 3796082 | 3796136 | 3796117 | 3796249 | 3795147 | 3796354 | 3795549 | 3799106 | 3801958 | 3805466 | 3802278 | 3805465 | 3805499 |
| # contigs | 22 | 22 | 23 | 23 | 23 | 23 | 23 | 22 | 20 | 21 | 22 | 19 | 19 | 18 | 20 | 21 |
| Largest contig | 896116 | 896117 | 896118 | 896120 | 896122 | 896124 | 896126 | 927497 | 927675 | 927760 | 927775 | 1184268 | 1184272 | 1184276 | 964517 | 964521 |
| Total length | 3798098 | 3798113 | 3798085 | 3800178 | 3800276 | 3800265 | 3800381 | 3799839 | 3800502 | 3799717 | 3803769 | 3806177 | 3808778 | 3805012 | 3809058 | 3809721 |
| GC (%) | 41.21 | 41.21 | 41.21 | 41.20 | 41.20 | 41.20 | 41.20 | 41.21 | 41.21 | 41.21 | 41.22 | 41.23 | 41.24 | 41.23 | 41.24 | 41.24 |
| N50 | 544317 | 544318 | 544319 | 544321 | 544323 | 544325 | 544327 | 876522 | 876526 | 876530 | 876534 | 951128 | 928339 | 928348 | 896205 | 896207 |
| N75 | 216424 | 216425 | 142494 | 142498 | 142502 | 142506 | 142510 | 216436 | 264688 | 216469 | 219713 | 896199 | 896201 | 896203 | 219713 | 219713 |
| L50 | 3 | 3 | 3 | 3 | 3 | 3 | 3 | 3 | 3 | 3 | 3 | 2 | 2 | 2 | 3 | 3 |
| L75 | 5 | 5 | 6 | 6 | 6 | 6 | 6 | 4 | 4 | 4 | 4 | 3 | 3 | 3 | 4 | 4 |
| # N's per 100 kbp | 16.98 | 16.85 | 23.67 | 23.13 | 22.60 | 25.63 | 25.02 | 21.00 | 19.60 | 12.34 | 9.25 | 13.58 | 11.95 | 11.77 | 10.13 | 10.05 |
| # contigs: number of contigs with a length ≥ 500pb.  Total lenght: number of bp in contigs with a length ≥ 500pb. | | | | | | | | | | | | | | | | |
