# Supplementary material for: De novo genome assembly of Bacillus altitudinis 19RS3 and Bacillus altitudinis T5S-T4, two plant growth-promoting bacteria isolated from Ilex paraguariensis St. Hil. (yerba mate)
Source: PLoS One. 2021 Mar 11;16(3):e0248274. doi: 10.1371/journal.pone.0248274 (PMC7954119; doi:10.1371/journal.pone.0248274)
Supplement: S2 Table — (DOCX) [file pone.0248274.s002.docx]

| **S2 Table.** Assembled genome quality statistics obtained for *Bacillus altitudinis* T5S-T4 a plant growth-promoting bacterium isolated from *Ilex paraguariensis* St. Hil. using ABySS assembler. | | | | | | | | | | | | | | | | |
| --- | --- | --- | --- | --- | --- | --- | --- | --- | --- | --- | --- | --- | --- | --- | --- | --- |
| Statistics | kmer 63 | kmer 65 | kmer 67 | kmer 69 | kmer 71 | kmer 73 | kmer 75 | kmer 77 | kmer 79 | kmer 81 | kmer 83 | kmer 85 | kmer 87 | kmer 89 | kmer 91 | kmer 95 |
| # contigs (>= 0 bp) | 262 | 232 | 235 | 216 | 206 | 208 | 199 | 174 | 168 | 156 | 154 | 138 | 129 | 126 | 112 | 101 |
| # contigs (>= 1000 bp) | 39 | 28 | 39 | 29 | 30 | 32 | 31 | 31 | 30 | 31 | 28 | 26 | 26 | 25 | 23 | 22 |
| Total length (>= 0 bp) | 3824635 | 3823710 | 3820134 | 3820664 | 3824459 | 3825053 | 3824041 | 3820759 | 3819127 | 3810726 | 3794351 | 3784936 | 3781416 | 3775919 | 3775652 | 3771411 |
| Total length (>= 1000 bp) | 3791416 | 3792272 | 3788883 | 3789271 | 3795113 | 3796803 | 3796011 | 3793603 | 3791594 | 3784524 | 3766457 | 3756738 | 3753524 | 3748251 | 3750057 | 3749365 |
| # contigs | 44 | 34 | 44 | 35 | 35 | 35 | 35 | 37 | 37 | 38 | 36 | 36 | 36 | 35 | 34 | 28 |
| Largest contig | 807219 | 807221 | 807223 | 807225 | 807227 | 807229 | 807255 | 807257 | 807259 | 807260 | 807260 | 807260 | 807260 | 807260 | 807260 | 807963 |
| Total length | 3794995 | 3796386 | 3792490 | 3793381 | 3798634 | 3798869 | 3798682 | 3797488 | 3796024 | 3789281 | 3772502 | 3763386 | 3760855 | 3755616 | 3757854 | 3753774 |
| GC (%) | 41.09 | 41.09 | 41.08 | 41.08 | 41.10 | 41.10 | 41.10 | 41.10 | 41.11 | 41.14 | 41.22 | 41.25 | 41.26 | 41.26 | 41.27 | 41.27 |
| N50 | 303986 | 446163 | 303994 | 551899 | 551901 | 551903 | 551905 | 551907 | 551909 | 551911 | 551961 | 551963 | 551965 | 552054 | 552056 | 552057 |
| N75 | 90212 | 145605 | 89395 | 145233 | 127127 | 127129 | 126996 | 126998 | 127115 | 126409 | 126411 | 126413 | 127116 | 127116 | 147509 | 182761 |
| L50 | 4 | 4 | 4 | 3 | 3 | 3 | 3 | 3 | 3 | 3 | 3 | 3 | 3 | 3 | 3 | 3 |
| L75 | 10 | 7 | 11 | 6 | 7 | 7 | 7 | 7 | 7 | 7 | 7 | 7 | 7 | 7 | 7 | 7 |
| # N's per 100 kbp | 6.35 | 27.82 | 0.21 | 17.98 | 17.66 | 16.77 | 15.03 | 9.01 | 11.99 | 7.34 | 15.96 | 5.34 | 4.76 | 2.66 | 3.99 | 5.49 |
| # contigs: number of contigs with a length ≥ 500pb.  Total lenght: number of bp in contigs with a length ≥ 500pb. | | | | | | | | | | | | | | | | |
