# Supplementary material for: De novo genome assembly of Bacillus altitudinis 19RS3 and Bacillus altitudinis T5S-T4, two plant growth-promoting bacteria isolated from Ilex paraguariensis St. Hil. (yerba mate)
Source: PLoS One. 2021 Mar 11;16(3):e0248274. doi: 10.1371/journal.pone.0248274 (PMC7954119; doi:10.1371/journal.pone.0248274)
Supplement: S3 Table — (DOCX) [file pone.0248274.s003.docx]

| **S3 Table.** Assembled genome quality statistics obtained for *Bacillus altitudinis* 19RS3 a plant growth-promoting bacterium isolated from *Ilex paraguariensis* St. Hil. using SPAdes assembler. | | | | | | | | | | | | | | |
| --- | --- | --- | --- | --- | --- | --- | --- | --- | --- | --- | --- | --- | --- | --- |
| Statistics | k-mer 55 | k-mer 61 | k-mer 65 | k-mer 67 | k-mer 69 | k-mer 71 | k-mer 73 | k-mer 75 | k-mer 77 | k-mer 79 | k-mer 81 | k-mer 83 | k-mer 85 | k-mer 87 |
| # contigs (>= 0 bp) | 177 | 159 | 146 | 142 | 125 | 146 | 107 | 109 | 71 | 53 | 54 | 52 | 52 | 57 |
| # contigs (>= 1000 bp) | 23 | 20 | 17 | 15 | 16 | 15 | 15 | 15 | 14 | 14 | 13 | 13 | 13 | 13 |
| Total length (>= 0 bp) | 3797134 | 3796149 | 3797065 | 3798960 | 3798123 | 3799913 | 3798126 | 3798546 | 3796124 | 3794786 | 3795943 | 3795957 | 3797955 | 3799514 |
| Total length (>= 1000 bp) | 3779408 | 3778360 | 3778972 | 3780788 | 3781748 | 3780576 | 3782758 | 3782107 | 3784918 | 3787263 | 3786757 | 3787024 | 3786063 | 3785587 |
| # contigs | 28 | 24 | 21 | 18 | 19 | 18 | 18 | 19 | 17 | 16 | 17 | 17 | 19 | 21 |
| Largest contig | 778854 | 830193 | 964992 | 964996 | 964386 | 964394 | 964402 | 964492 | 966288 | 966271 | 964864 | 964870 | 964876 | 964882 |
| Total length | 3782731 | 3781196 | 3781853 | 3783031 | 3783999 | 3782835 | 3785025 | 3784944 | 3787201 | 3788682 | 3789487 | 3789762 | 3790302 | 3791338 |
| GC (%) | 41.16 | 41.16 | 41.16 | 41.16 | 41.16 | 41.16 | 41.16 | 41.16 | 41.17 | 41.18 | 41.18 | 41.18 | 41.18 | 41.18 |
| N50 | 553021 | 774421 | 927810 | 927814 | 927818 | 927786 | 928507 | 928507 | 929616 | 931914 | 932026 | 932030 | 930818 | 894990 |
| N75 | 154531 | 236800 | 337002 | 348320 | 348324 | 348328 | 337095 | 337095 | 348340 | 348459 | 348463 | 348467 | 348638 | 348685 |
| L50 | 3 | 3 | 2 | 2 | 2 | 2 | 2 | 2 | 2 | 2 | 2 | 2 | 2 | 3 |
| L75 | 6 | 5 | 4 | 4 | 4 | 4 | 4 | 4 | 4 | 4 | 4 | 4 | 4 | 4 |
| # N's per 100 kbp | 0.00 | 5.29 | 2.64 | 2.64 | 5.29 | 5.29 | 5.28 | 5.28 | 2.64 | 0.00 | 5.28 | 5.28 | 5.28 | 7.68 |
| # contigs: number of contigs with a length ≥ 500pb.  Total lenght: number of bp in contigs with a length ≥ 500pb. | | | | | | | | | | | | | | |
