# Supplementary material for: De novo genome assembly of Bacillus altitudinis 19RS3 and Bacillus altitudinis T5S-T4, two plant growth-promoting bacteria isolated from Ilex paraguariensis St. Hil. (yerba mate)
Source: PLoS One. 2021 Mar 11;16(3):e0248274. doi: 10.1371/journal.pone.0248274 (PMC7954119; doi:10.1371/journal.pone.0248274)
Supplement: S4 Table — (DOCX) [file pone.0248274.s004.docx]

| **S4 Table.** Assembled genome quality statistics obtained for *Bacillus altitudinis* T5S-T4 a plant growth-promoting bacterium isolated from *Ilex paraguariensis* St. Hil. using SPAdes assembler. | | | | | | | | | | | | | | | | |
| --- | --- | --- | --- | --- | --- | --- | --- | --- | --- | --- | --- | --- | --- | --- | --- | --- |
| Statistics | k-mer 55 | k-mer 61 | k-mer 71 | k-mer 73 | k-mer 75 | k-mer 77 | k-mer 79 | k-mer 81 | k-mer 83 | k-mer 85 | k-mer 87 | k-mer 89 | k-mer 91 | k-mer 93 | k-mer 95 | k-mer 97 |
| # contigs (>= 0 bp) | 929 | 693 | 833 | 417 | 383 | 354 | 332 | 84 | 87 | 93 | 371 | 126 | 159 | 82 | 57 | 45 |
| # contigs (>= 1000 bp) | 100 | 99 | 85 | 83 | 94 | 117 | 153 | 37 | 35 | 37 | 46 | 35 | 36 | 33 | 30 | 31 |
| Total length (>= 0 bp) | 6406755 | 6383833 | 6405122 | 6371535 | 6366976 | 6357061 | 6340490 | 3784961 | 3787062 | 3791969 | 3907081 | 3804060 | 3813498 | 3765452 | 3743490 | 3737914 |
| Total length (>= 1000 bp) | 6310507 | 6313840 | 6320119 | 6322062 | 6316813 | 6314130 | 6300535 | 3769522 | 3768484 | 3770937 | 3787010 | 3773354 | 3771663 | 3747258 | 3733516 | 3733005 |
| # contigs | 115 | 113 | 96 | 96 | 114 | 134 | 170 | 48 | 49 | 53 | 115 | 49 | 53 | 44 | 37 | 34 |
| Largest contig | 805045 | 805057 | 805573 | 805081 | 805109 | 805113 | 805117 | 805121 | 805125 | 805129 | 805133 | 805137 | 805141 | 805145 | 805149 | 805153 |
| Total length | 6320832 | 6323523 | 6327512 | 6330982 | 6330686 | 6325232 | 6312828 | 3777671 | 3779066 | 3782326 | 3834109 | 3783572 | 3783282 | 3755035 | 3738539 | 3734963 |
| GC (%) | 38.02 | 38.02 | 38.02 | 38.03 | 38.03 | 38.03 | 38.05 | 41.10 | 41.11 | 41.11 | 41.03 | 41.12 | 41.12 | 41.20 | 41.24 | 41.24 |
| N50 | 159588 | 179004 | 181370 | 181374 | 181378 | 123675 | 123679 | 344076 | 344080 | 344084 | 344088 | 344092 | 344096 | 344100 | 318456 | 344108 |
| N75 | 62410 | 63310 | 81024 | 80933 | 81116 | 49896 | 39253 | 125713 | 125717 | 125721 | 125866 | 179163 | 179167 | 179171 | 179175 | 179103 |
| L50 | 10 | 9 | 9 | 9 | 10 | 11 | 11 | 4 | 4 | 4 | 4 | 4 | 4 | 4 | 4 | 4 |
| L75 | 27 | 24 | 23 | 23 | 24 | 32 | 38 | 8 | 8 | 8 | 8 | 7 | 7 | 7 | 8 | 7 |
| # N's per 100 kbp | 3.16 | 1.74 | 2.75 | 3.47 | 7.41 | 30.37 | 88.79 | 0.00 | 0.00 | 0.00 | 38.13 | 18.58 | 28.28 | 25.33 | 6.58 | 9.32 |
| # contigs: number of contigs with a length ≥ 500pb.  Total lenght: number of bp in contigs with a length ≥ 500pb. | | | | | | | | | | | | | | | | |
