# Supplementary material for: De novo genome assembly of Bacillus altitudinis 19RS3 and Bacillus altitudinis T5S-T4, two plant growth-promoting bacteria isolated from Ilex paraguariensis St. Hil. (yerba mate)
Source: PLoS One. 2021 Mar 11;16(3):e0248274. doi: 10.1371/journal.pone.0248274 (PMC7954119; doi:10.1371/journal.pone.0248274)
Supplement: S5 Table — (DOCX) [file pone.0248274.s005.docx]

| **S5 Table.** Assembled genome quality statistics obtained for *Bacillus altitudinis* 19RS3 a plant growth-promoting bacterium isolated from *Ilex paraguariensis* St. Hil. using Velvet assembler. | | | | | | | | | | | | | | | | |
| --- | --- | --- | --- | --- | --- | --- | --- | --- | --- | --- | --- | --- | --- | --- | --- | --- |
| Statistics | k-mer 63 | k-mer 65 | k-mer 67 | k-mer 69 | k-mer 71 | k-mer 73 | k-mer 75 | k-mer 77 | k-mer 79 | k-mer 81 | k-mer 83 | k-mer 85 | k-mer 87 | k-mer 89 | k-mer 91 | k-mer 93 |
| # contigs (>= 0 bp) | 45 | 47 | 48 | 48 | 47 | 47 | 44 | 43 | 40 | 38 | 36 | 37 | 32 | 27 | 28 | 26 |
| # contigs (>= 1000 bp) | 13 | 14 | 13 | 13 | 13 | 14 | 12 | 12 | 14 | 12 | 13 | 13 | 12 | 13 | 13 | 12 |
| Total length (>= 0 bp) | 3794721 | 3794627 | 3795860 | 3796267 | 3796347 | 3796532 | 3796025 | 3795442 | 3794538 | 3794491 | 3794692 | 3794768 | 3794359 | 3793521 | 3794508 | 3794712 |
| Total length (>= 1000 bp) | 3786924 | 3786252 | 3787070 | 3787391 | 3787406 | 3788007 | 3786275 | 3785697 | 3785752 | 3785606 | 3786263 | 3786147 | 3786976 | 3788397 | 3789046 | 3789435 |
| # contigs | 17 | 18 | 17 | 17 | 17 | 18 | 18 | 18 | 19 | 18 | 19 | 19 | 17 | 16 | 16 | 15 |
| Largest contig | 976456 | 966265 | 976338 | 976684 | 976512 | 974063 | 976651 | 976228 | 966318 | 975865 | 976618 | 976229 | 1012274 | 1012311 | 1004514 | 1943801 |
| Total length | 3789740 | 3789080 | 3789910 | 3790243 | 3790270 | 3790730 | 3790607 | 3790263 | 3789610 | 3790188 | 3790939 | 3790745 | 3791010 | 3790842 | 3791499 | 3791896 |
| GC (%) | 41.18 | 41.18 | 41.18 | 41.18 | 41.18 | 41.18 | 41.18 | 41.18 | 41.18 | 41.18 | 41.18 | 41.18 | 41.18 | 41.18 | 41.18 | 41.18 |
| N50 | 966285 | 929008 | 966217 | 966271 | 966248 | 966316 | 966295 | 966266 | 929975 | 966312 | 966460 | 966463 | 1004598 | 1004513 | 976774 | 1943801 |
| N75 | 337677 | 337684 | 337692 | 337699 | 337707 | 337714 | 337712 | 337691 | 337697 | 337703 | 263293 | 263524 | 930805 | 930745 | 966847 | 1004695 |
| L50 | 2 | 2 | 2 | 2 | 2 | 2 | 2 | 2 | 2 | 2 | 2 | 2 | 2 | 2 | 2 | 1 |
| L75 | 4 | 4 | 4 | 4 | 4 | 4 | 4 | 4 | 4 | 4 | 4 | 4 | 3 | 3 | 3 | 2 |
| # N's per 100 kbp | 83.88 | 59.94 | 72.38 | 51.03 | 76.04 | 64.02 | 57.67 | 33.35 | 28.42 | 26.23 | 40.25 | 35.48 | 29.28 | 14.35 | 31.76 | 39.98 |
| # contigs: number of contigs with a length ≥ 500pb.  Total lenght: number of bp in contigs with a length ≥ 500pb. | | | | | | | | | | | | | | | | |
