# Supplementary material for: De novo genome assembly of Bacillus altitudinis 19RS3 and Bacillus altitudinis T5S-T4, two plant growth-promoting bacteria isolated from Ilex paraguariensis St. Hil. (yerba mate)
Source: PLoS One. 2021 Mar 11;16(3):e0248274. doi: 10.1371/journal.pone.0248274 (PMC7954119; doi:10.1371/journal.pone.0248274)
Supplement: S6 Table — (DOCX) [file pone.0248274.s006.docx]

| **S6 Table.** Assembled genome quality statistics obtained for *Bacillus altitudinis* T5S-T4 a plant growth-promoting bacterium isolated from *Ilex paraguariensis* St. Hil. using Velvet assembler. | | | | | | | | | | | | | | | | |
| --- | --- | --- | --- | --- | --- | --- | --- | --- | --- | --- | --- | --- | --- | --- | --- | --- |
| Statistics | k-mer 63 | k-mer 65 | k-mer 67 | k-mer 69 | k-mer 71 | k-mer 73 | k-mer 75 | k-mer 77 | k-mer 79 | k-mer 81 | k-mer 83 | k-mer 85 | k-mer 87 | k-mer 89 | k-mer 91 | k-mer 93 |
| # contigs (>= 0 bp) | 54 | 52 | 55 | 52 | 52 | 53 | 49 | 54 | 56 | 56 | 47 | 51 | 41 | 43 | 43 | 46 |
| # contigs (>= 1000 bp) | 27 | 26 | 25 | 26 | 25 | 27 | 27 | 27 | 27 | 28 | 27 | 26 | 23 | 23 | 24 | 24 |
| Total length (>= 0 bp) | 3737416 | 3739318 | 3740374 | 3739825 | 3739713 | 3739917 | 3740873 | 3739851 | 3738406 | 3738628 | 3737261 | 3738565 | 3737966 | 3738127 | 3737448 | 3738095 |
| Total length (>= 1000 bp) | 3730672 | 3732707 | 3732053 | 3732666 | 3732363 | 3733580 | 3735089 | 3732873 | 3730189 | 3731277 | 3731841 | 3730987 | 3732630 | 3732140 | 3731590 | 3731486 |
| # contigs | 29 | 28 | 29 | 28 | 27 | 27 | 28 | 29 | 29 | 29 | 27 | 28 | 24 | 24 | 25 | 25 |
| Largest contig | 827175 | 805065 | 805067 | 805052 | 805075 | 805079 | 805166 | 805111 | 805115 | 805119 | 805123 | 805127 | 805131 | 805135 | 805139 | 805221 |
| Total length | 3732078 | 3734115 | 3734635 | 3734223 | 3733922 | 3733580 | 3735662 | 3734061 | 3731518 | 3731876 | 3731841 | 3732584 | 3733339 | 3732853 | 3732307 | 3732207 |
| GC (%) | 41.23 | 41.24 | 41.24 | 41.24 | 41.23 | 41.24 | 41.24 | 41.23 | 41.24 | 41.23 | 41.23 | 41.24 | 41.23 | 41.23 | 41.23 | 41.23 |
| N50 | 395831 | 344042 | 395840 | 344050 | 344054 | 344058 | 344062 | 344066 | 344070 | 344074 | 344078 | 344138 | 344145 | 344151 | 344157 | 344098 |
| N75 | 145375 | 179070 | 181360 | 179078 | 181368 | 179086 | 181376 | 181380 | 129602 | 155693 | 181392 | 181396 | 296513 | 315798 | 181408 | 181561 |
| L50 | 3 | 4 | 3 | 4 | 4 | 4 | 4 | 4 | 4 | 4 | 4 | 4 | 4 | 4 | 4 | 4 |
| L75 | 7 | 8 | 6 | 7 | 7 | 7 | 7 | 7 | 8 | 8 | 7 | 7 | 6 | 6 | 7 | 7 |
| # N's per 100 kbp | 50.24 | 69.12 | 72.38 | 69.79 | 74.61 | 55.60 | 75.19 | 58.41 | 34.62 | 28.73 | 28.62 | 25.99 | 28.82 | 21.38 | 22.40 | 46.84 |
| # contigs: number of contigs with a length ≥ 500pb.  Total lenght: number of bp in contigs with a length ≥ 500pb. | | | | | | | | | | | | | | | | |
