# Supplementary material for: De novo genome assembly of Bacillus altitudinis 19RS3 and Bacillus altitudinis T5S-T4, two plant growth-promoting bacteria isolated from Ilex paraguariensis St. Hil. (yerba mate)
Source: PLoS One. 2021 Mar 11;16(3):e0248274. doi: 10.1371/journal.pone.0248274 (PMC7954119; doi:10.1371/journal.pone.0248274)
Supplement: S7 Table — (DOCX) [file pone.0248274.s007.docx]

| **S7 Table.** Assembled genome quality statistics obtained for *Bacillus altitudinis* 19RS3 a plant growth-promoting bacterium isolated from *Ilex paraguariensis* St. Hil. using SOAPdenovo2 assembler. | | | | | | | | | | | | | | | |
| --- | --- | --- | --- | --- | --- | --- | --- | --- | --- | --- | --- | --- | --- | --- | --- |
| Statistics | k-mer 63 | k-mer 65 | k-mer 67 | k-mer 69 | k-mer 71 | k-mer 73 | k-mer 75 | k-mer 77 | k-mer 79 | k-mer 81 | k-mer 83 | k-mer 85 | k-mer 87 | k-mer 89 | k-mer 91 |
| # contigs (>= 0 bp) | 373 | 339 | 313 | 295 | 286 | 278 | 255 | 233 | 220 | 206 | 200 | 187 | 172 | 162 | 158 |
| # contigs (>= 1000 bp) | 86 | 82 | 81 | 74 | 72 | 68 | 64 | 57 | 52 | 46 | 44 | 40 | 39 | 39 | 41 |
| Total length (>= 0 bp) | 3834883 | 3831303 | 3826350 | 3823318 | 3821357 | 3818892 | 3815972 | 3812694 | 3810206 | 3808607 | 3808139 | 3806327 | 3804358 | 3803118 | 3802490 |
| Total length (>= 1000 bp) | 3770892 | 3772765 | 3774758 | 3774133 | 3774231 | 3773374 | 3775486 | 3776975 | 3776797 | 3776756 | 3777940 | 3777918 | 3777985 | 3778204 | 3778586 |
| # contigs | 107 | 102 | 100 | 95 | 89 | 82 | 76 | 64 | 60 | 54 | 50 | 45 | 44 | 43 | 45 |
| Largest contig | 347517 | 347519 | 347521 | 347523 | 347525 | 347527 | 347529 | 347531 | 453045 | 453049 | 453053 | 453057 | 492822 | 492824 | 575375 |
| Total length | 3785130 | 3786694 | 3787623 | 3787506 | 3785284 | 3782702 | 3783472 | 3782207 | 3782594 | 3782736 | 3782046 | 3781428 | 3781507 | 3781234 | 3781628 |
| GC (%) | 41.17 | 41.18 | 41.17 | 41.17 | 41.17 | 41.16 | 41.16 | 41.16 | 41.16 | 41.16 | 41.16 | 41.16 | 41.16 | 41.16 | 41.16 |
| N50 | 66124 | 67072 | 87944 | 93759 | 93763 | 95544 | 109297 | 134561 | 144518 | 152698 | 164162 | 227671 | 227673 | 227675 | 250230 |
| N75 | 43706 | 44920 | 48096 | 49740 | 52804 | 54640 | 55590 | 66152 | 67038 | 68161 | 86059 | 88189 | 93796 | 93800 | 82983 |
| L50 | 16 | 15 | 14 | 13 | 13 | 12 | 12 | 10 | 8 | 8 | 8 | 6 | 6 | 6 | 5 |
| L75 | 33 | 32 | 30 | 28 | 27 | 26 | 24 | 20 | 18 | 17 | 16 | 13 | 12 | 12 | 12 |
| # N's per 100 kbp | 0.00 | 0.00 | 0.00 | 0.00 | 0.00 | 0.00 | 0.00 | 0.00 | 0.00 | 0.00 | 0.00 | 0.00 | 0.00 | 0.00 | 0.00 |
| # contigs: number of contigs with a length ≥ 500pb.  Total lenght: number of bp in contigs with a length ≥ 500pb. | | | | | | | | | | | | | | | |
