# Supplementary material for: De novo genome assembly of Bacillus altitudinis 19RS3 and Bacillus altitudinis T5S-T4, two plant growth-promoting bacteria isolated from Ilex paraguariensis St. Hil. (yerba mate)
Source: PLoS One. 2021 Mar 11;16(3):e0248274. doi: 10.1371/journal.pone.0248274 (PMC7954119; doi:10.1371/journal.pone.0248274)
Supplement: S8 Table — (DOCX) [file pone.0248274.s008.docx]

| **S8 Table.** Assembled genome quality statistics obtained for *Bacillus altitudinis* T5S-T4 a plant growth-promoting bacterium isolated from *Ilex paraguariensis* St. Hil. using SOAPdenovo2 assembler. | | | | | | | | | | | | | | | | |
| --- | --- | --- | --- | --- | --- | --- | --- | --- | --- | --- | --- | --- | --- | --- | --- | --- |
| Statistics | k-mer 63 | k-mer 65 | k-mer 67 | k-mer 69 | k-mer 71 | k-mer 73 | k-mer 75 | k-mer 77 | k-mer 79 | k-mer 81 | k-mer 83 | k-mer 85 | k-mer 87 | k-mer 89 | k-mer 91 | k-mer 93 |
| # contigs (>= 0 bp) | 1717 | 2087 | 2570 | 3215 | 3864 | 4588 | 5227 | 5646 | 5580 | 4809 | 3349 | 1744 | 700 | 281 | 211 | 204 |
| # contigs (>= 1000 bp) | 870 | 971 | 1065 | 1038 | 940 | 743 | 474 | 245 | 124 | 74 | 68 | 66 | 54 | 52 | 51 | 74 |
| Total length (>= 0 bp) | 6413584 | 6427199 | 6443635 | 6457662 | 6457925 | 6428190 | 6344941 | 6168165 | 5866835 | 5403827 | 4806901 | 4257674 | 3921930 | 3790504 | 3764054 | 3757008 |
| Total length (>= 1000 bp) | 6099296 | 5963967 | 5774020 | 5445891 | 5097092 | 4680468 | 4267259 | 3953370 | 3809830 | 3755666 | 3740457 | 3736346 | 3725867 | 3724944 | 3723803 | 3725564 |
| # contigs | 1120 | 1362 | 1649 | 1928 | 2081 | 2094 | 1890 | 1526 | 1009 | 513 | 229 | 126 | 85 | 71 | 61 | 82 |
| Largest contig | 181354 | 181358 | 181362 | 211987 | 211991 | 211995 | 246701 | 255118 | 255122 | 255126 | 262621 | 262814 | 656689 | 656863 | 656867 | 411703 |
| Total length | 6277075 | 6241590 | 6188630 | 6075066 | 5898545 | 5620419 | 5242241 | 4810346 | 4377320 | 4029339 | 3838916 | 3774729 | 3746921 | 3737414 | 3730638 | 3731427 |
| GC (%) | 38.03 | 38.06 | 38.10 | 38.20 | 38.34 | 38.58 | 38.96 | 39.47 | 40.07 | 40.65 | 40.98 | 41.12 | 41.19 | 41.22 | 41.23 | 41.23 |
| N50 | 27824 | 28079 | 33819 | 35617 | 36859 | 41513 | 55696 | 68565 | 92553 | 106802 | 123630 | 123634 | 148351 | 148355 | 131452 | 85976 |
| N75 | 4404 | 3299 | 2427 | 1881 | 1578 | 1382 | 1405 | 12728 | 35516 | 49346 | 63647 | 63651 | 81140 | 81148 | 92577 | 48820 |
| L50 | 47 | 43 | 40 | 37 | 35 | 31 | 24 | 19 | 15 | 12 | 10 | 10 | 7 | 7 | 8 | 13 |
| L75 | 240 | 283 | 339 | 381 | 397 | 344 | 184 | 53 | 35 | 24 | 20 | 20 | 16 | 16 | 16 | 27 |
| # N's per 100 kbp | 0.00 | 0.00 | 0.00 | 0.00 | 0.00 | 0.00 | 0.00 | 0.00 | 0.00 | 0.00 | 0.00 | 0.00 | 0.00 | 0.00 | 0.00 | 0.00 |
| # contigs: number of contigs with a length ≥ 500pb.  Total lenght: number of bp in contigs with a length ≥ 500pb. | | | | | | | | | | | | | | | | |
