# Supplementary material for: De novo genome assembly of Bacillus altitudinis 19RS3 and Bacillus altitudinis T5S-T4, two plant growth-promoting bacteria isolated from Ilex paraguariensis St. Hil. (yerba mate)
Source: PLoS One. 2021 Mar 11;16(3):e0248274. doi: 10.1371/journal.pone.0248274 (PMC7954119; doi:10.1371/journal.pone.0248274)
Supplement: S9 Table — (DOCX) [file pone.0248274.s009.docx]

| **S9 Table.** Assembled genome quality statistics obtained for *Bacillus altitudinis* 19RS3 a plant growth-promoting bacterium isolated from *Ilex paraguariensis* St. Hil. using Geneious assembler with the Velvet algorithm. | | | | | | | | | | | | | | | |
| --- | --- | --- | --- | --- | --- | --- | --- | --- | --- | --- | --- | --- | --- | --- | --- |
| Statistics | k-mer 63 | k-mer 65 | k-mer 67 | k-mer 69 | k-mer 71 | k-mer 73 | k-mer 75 | k-mer 77 | k-mer 79 | k-mer 81 | k-mer 83 | k-mer 85 | k-mer 87 | k-mer 89 | k-mer 91 |
| Contigs >=1000 bp | 65 | 62 | 62 | 62 | 59 | 53 | 54 | 50 | 48 | 45 | 44 | 42 | 40 | 37 | 37 |
| Min Length (bp) | 1,006 | 1,008 | 1,010 | 1,012 | 1,014 | 1,016 | 1,018 | 1,020 | 1,022 | 1,024 | 1,026 | 1,028 | 1,030 | 1,032 | 1,034 |
| Median Length (bp) | 47,131 | 48,130 | 46,646 | 45,306 | 49,114 | 46,169 | 45,178 | 50,612 | 49,531 | 50,726 | 51,651 | 51,655 | 63,017 | 66,466 | 62,246 |
| Mean Length (bp) | 58,025 | 60,844 | 60,849 | 60,863 | 63,962 | 71,189 | 69,932 | 75,538 | 78,733 | 84,019 | 85,949 | 90,039 | 94,568 | 102,322 | 102,309 |
| Max Length (bp) | 440,394 | 440,402 | 440,410 | 440,499 | 353,091 | 440,880 | 440,884 | 440,888 | 440,892 | 494,050 | 494,056 | 765,098 | 549,780 | 580,443 | 563,940 |
| N50 Length (bp) | 87,896 | 98,911 | 105,645 | 105,649 | 111,043 | 134,551 | 134,555 | 144,512 | 152,692 | 154,442 | 167,993 | 167,997 | 168,001 | 205,251 | 222,742 |
| Number of contigs >= N50 | 13 | 12 | 12 | 12 | 12 | 9 | 9 | 9 | 8 | 7 | 7 | 6 | 7 | 6 | 6 |
| Length Sum (bp) | 3,771,684 | 3,772,361 | 3,772,682 | 3,773,552 | 3,773,760 | 3,773,033 | 3,776,365 | 3,776,904 | 3,779,193 | 3,780,859 | 3,781,768 | 3,781,675 | 3,782,720 | 3,785,917 | 3,785,433 |
