# Supplementary material for: De novo genome assembly of Bacillus altitudinis 19RS3 and Bacillus altitudinis T5S-T4, two plant growth-promoting bacteria isolated from Ilex paraguariensis St. Hil. (yerba mate)
Source: PLoS One. 2021 Mar 11;16(3):e0248274. doi: 10.1371/journal.pone.0248274 (PMC7954119; doi:10.1371/journal.pone.0248274)
Supplement: S10 Table — (DOCX) [file pone.0248274.s010.docx]

| **S10 Table.** Assembled genome quality statistics obtained for *Bacillus altitudinis* T5S-T4 a plant growth-promoting bacterium isolated from *Ilex paraguariensis* St. Hil. using Geneious assembler with the Velvet algorithm. | | | | | | | | | | | | | | | |
| --- | --- | --- | --- | --- | --- | --- | --- | --- | --- | --- | --- | --- | --- | --- | --- |
| Statistics | k-mer 63 | k-mer 65 | k-mer 67 | k-mer 69 | k-mer 71 | k-mer 73 | k-mer 75 | k-mer 77 | k-mer 79 | k-mer 81 | k-mer 83 | k-mer 85 | k-mer 87 | k-mer 89 | k-mer 91 |
| Contigs >=1000 bp | 68 | 64 | 64 | 65 | 62 | 62 | 58 | 56 | 55 | 52 | 52 | 52 | 46 | 44 | 45 |
| Min Length (bp) | 1,058 | 1,064 | 1,077 | 1,169 | 1,173 | 1,177 | 1,181 | 1,185 | 1,286 | 1,177 | 1,007 | 1,011 | 1,286 | 1,286 | 1,286 |
| Median Length (bp) | 39,176 | 38,141 | 38,145 | 36,215 | 40,415 | 40,427 | 41,975 | 43,294 | 43,402 | 41,394 | 38,219 | 36,714 | 41,648 | 41,412 | 39,275 |
| Mean Length (bp) | 54,634 | 58,055 | 58,104 | 57,222 | 59,998 | 60,027 | 64,134 | 66,445 | 67,703 | 71,584 | 71,695 | 71,703 | 81,081 | 84,774 | 82,897 |
| Max Length (bp) | 197,863 | 211,977 | 211,981 | 211,987 | 211,991 | 211,995 | 246,699 | 246,703 | 255,120 | 255,124 | 391,162 | 487,918 | 487,922 | 656,691 | 656,865 |
| N50 Length (bp) | 97,136 | 97,374 | 104,818 | 97,901 | 97,412 | 97,683 | 104,834 | 104,838 | 117,661 | 143,588 | 148,341 | 154,792 | 155,378 | 155,382 | 155,386 |
| Number of contigs >= N50 | 13 | 13 | 12 | 13 | 13 | 13 | 12 | 12 | 11 | 10 | 9 | 8 | 7 | 6 | 6 |
| Length Sum (bp) | 3,715,145 | 3,715,575 | 3,718,667 | 3,719,448 | 3,719,892 | 3,721,681 | 3,719,784 | 3,720,927 | 3,723,705 | 3,722,396 | 3,728,183 | 3,728,599 | 3,729,742 | 3,730,065 | 3,730,409 |
