# Supplementary material for: De novo genome assembly of Bacillus altitudinis 19RS3 and Bacillus altitudinis T5S-T4, two plant growth-promoting bacteria isolated from Ilex paraguariensis St. Hil. (yerba mate)
Source: PLoS One. 2021 Mar 11;16(3):e0248274. doi: 10.1371/journal.pone.0248274 (PMC7954119; doi:10.1371/journal.pone.0248274)
Supplement: S11 Table — (DOCX) [file pone.0248274.s011.docx]

| **S11 Table.** Assembled genome quality statistics obtained for *Bacillus altitudinis* 19RS3 a plant growth-promoting bacterium isolated from *Ilex paraguariensis* St. Hil. using CLC Workbench assembler. | | | | |
| --- | --- | --- | --- | --- |
| Statistics | Automatic word size (20) | Automatic word size (20) | Word-size 64 | Word-size 64 |
| Minimum contig lenght | 500 pb | 1000 pb | 500 pb | 1000 pb |
| N75 | 119.573 | 215.215 | 215.217 | 262.922 |
| N50 | 520.901 | 795.157 | 895.161 | 928.728 |
| N25 | 773.786 | 866.607 | 966.324 | 966.324 |
| Minimum | 507 | 1034 | 523 | 1360 |
| Maximum | 787.120 | 866.607 | 966.324 | 966.324 |
| Average | 12.670 | 39.401 | 88.473 | 210.387 |
| # contigs | 308 | 97 | 43 | 18 |
| Total reads | 3.902.480 | 3.821.923 | 3.804.336 | 3.786.967 |
