# Supplementary material for: De novo genome assembly of Bacillus altitudinis 19RS3 and Bacillus altitudinis T5S-T4, two plant growth-promoting bacteria isolated from Ilex paraguariensis St. Hil. (yerba mate)
Source: PLoS One. 2021 Mar 11;16(3):e0248274. doi: 10.1371/journal.pone.0248274 (PMC7954119; doi:10.1371/journal.pone.0248274)
Supplement: S12 Table — (DOCX) [file pone.0248274.s012.docx]

| **S12 Table.** Assembled genome quality statistics obtained for *Bacillus altitudinis* T5S-T4 a plant-growth-promoting bacteria isolated from *Ilex paraguariensis* St. Hil. using CLC Workbench assembler. | | | | |
| --- | --- | --- | --- | --- |
| Statistics | Automatic word size (20) | Automatic word size (20) | Word-size 64 | Word-size 64 |
| Minimum contig lenght | 500 pb | 1000 pb | 500pb | 1000pb |
| N75 | 123.989 | 117.387 | 88.137 | 88.137 |
| N50 | 214.550 | 178.964 | 141.754 | 141.753 |
| N25 | 357.548 | 357.535 | 357.698 | 357.698 |
| Minimum | 506 | 1180 | 510 | 1032 |
| Maximum | 534.586 | 534.675 | 805.085 | 805.085 |
| Average | 80.276 | 85.618 | 68.171 | 77.237 |
| # contigs | 79 | 74 | 93 | 82 |
| Total reads | 6.341.839 | 6.335.788 | 6.339.908 | 6.333.395 |
