# Supplementary material for: De novo genome assembly of Bacillus altitudinis 19RS3 and Bacillus altitudinis T5S-T4, two plant growth-promoting bacteria isolated from Ilex paraguariensis St. Hil. (yerba mate)
Source: PLoS One. 2021 Mar 11;16(3):e0248274. doi: 10.1371/journal.pone.0248274 (PMC7954119; doi:10.1371/journal.pone.0248274)
Supplement: Attachment — Submitted filename: Response to Reviewers.docx [file pone.0248274.s013.docx]

**Response to reviewers**

**Title: De novo genome assembly of *Bacillus altitudinis* 19RS3 and *Bacillus altitudinis* T5S-T4, two Plant Growth-Promoting Bacteria isolated from *Ilex paraguariensis* St. Hil. (yerba mate)**

**Authors: Iliana Julieta Cortese, María Lorena Castrillo, Andrea Liliana Onetto, Gustavo Ángel Bich, Pedro Darío Zapata, Margarita Ester Laczeski.**

First, we want to express our sincere thanks to the reviewers for their great work, their notes have allowed us not only to improve the manuscript but also to reflect on future research.

After a careful review of our proposed article, based on the suggestions made by the reviewers, we have processed your submission for a new evaluation. In the new manuscript, we have used the track changes mode in Word for the modifications made to the original text as you suggested.

We hope that the work done will achieve the final approval of the Editorial Team. If not, all authors are available to resolve any issue or proceed with further revisions, as necessary.

We then respond to each of the reviewers' observations.

Reviewer Comments:

**Reviewer 1**

*This manuscript describes the de novo sequencing and assembly of genomes from two isolates of Bacillus altitudinis that were recovered from plant tissue. The authors have used various computational tools to obtain the best assembly,and have annotated the genomes of both isolates. Part of the annotation identified putative functions that may be related to the plant growth promotion capabilities of the isolates. The sequencing and analysis is pretty routine, but the results are pretty clear and this provides helpful information to the body of information of genome analysis of plant growth promoting rhizobacteria (PGPR). I have two important points to be addressed, plus a few minor comments, as follows:*

*1. The paper would be strengthened by examining the relationship between these two new genomes with other published Bacillus genomes, especially those with PGPR activity. When compared, what genes are conserved or are novel? This would be helpful to know in order to more closely connect potential genes with PGPR mechanisms*

Response: Following your suggestion we have incorporated in the manuscript the conserved genes of both bacterial strains related to PGP properties.

*2. Related to the above, the current analysis identifies broad categories of genes potentially involved in PGPR activity, such as iron metabolism, but some more in depth analysis of specific genes would be helpful. In particular, identify if genes are present that have been identified in other Bacillus species.*

Response: As suggested, new relationships between these two new genomes and other published *Bacillus* genomes, especially of bacteria with PGPR activity, were examined. A table was added to add information and improve analysis in the results section. In addition, related information was added to the discussion.

*3. Line 130, what tissues were used for DNA isolation? Leaves, etc.?*

Response: We changed tissues for roots.

*4. Please have someone carefully proofread for proper English, including use of 'a', 'an', and 'the'. Often one of these words is present when not needed, or absent when it is needed. See for example in lines 38, 73 and 86.*

Response: English proofread was checked

*5. Line 32 should read 'Assembly evaluation was done...'*

Response: We changed the text.

*6. line 66, should read '...great diversity of species...', 'Kepping' should be 'Keeping'*

Response: We corrected the word.

*7. line 68, 'perspective' seems to be the wrong word here.*

Response: The expression has been improved.

*8. line 82, should be 'revealed'*

Response: We changed the text.

*9. lines 105, 106, remove 's' from the ends of words where it is not needed.*

Response: We removed “s” from the ends of words where it wasn´t needed.

*10. line 142, 'in' should be 'by'*

Response: We changed the word.

*11. line 176, 'bigger' is an awkward word here, better to say 'higher' or 'larger'*

Response: We change bigger to higher.

*12. lines 181-183, the end of the sentence after the last comma is redundant and should be removed.*

Response: We removed the sentence.

*13. line 190 and table, are the accession numbers really all zeros after the initial letters, or are these placeholders to be updated?*

Response: We thank the reviewer for this particularly important remark. We corrected the accession numbers of both genomes.

**Reviewer 2**

*General comments: This manuscript described the assembly and annotation of two plant growth-promoting bacteria. The authors describe multiple different assemblies constructed using various different software programs and choose the assembly they believe is the best. This is an interesting dataset and reporting that could be of interest to bacterial researchers and researchers interested in using more natural means of beneficials for plant growth and overall health.*

*1. One main concern of the manuscript is what this adds to the community. Many previous studies have looked at bacterial genome assemblies across multiple software types. It seems for the most part this manuscript agrees with basically all previous findings. The authors should really focus and point out what their results are adding to the community. Adding the assemblies of PGPB is great and justified, but the manuscript focuses so much on the assembly of multiple softwares that it dilutes down the importance of having these genomes without really adding much to the space of assembly software decisions.*

Response: We thank the Reviewer 2 for her/his careful reading of the manuscript and for her/his constructive remarks, which were useful in improving our paper. We followed this important comment. In the new version of our manuscript, we added more information about the PGP genes detected in both genomes. We considered this information should be useful for future comparative genome analyses to provide a better understanding of beneficial plant-bacteria associations.

*2. The methods are insufficient in details. It would be really difficult for anyone to reproduce your results and assemblies. We are not told the types of reads used, how each of the assembly software was run and utilized (what parameters were used or changed or prioritized).*

Response: We added the type of reads used. About the parameters applied for each software we used the pre-determinate options indicated in the manual user instructions and we only vary the k-mer value. In the new version of our manuscript, we include the genome quality statistics generated by different assemblers as Supplementary material.

*3. Something isn’t right with the T5S-T4 CLC workbench results in Table 3. The largest contain is 178bp but the N50 is 895bp?*

Response: We checked and corrected the values.

*4. I’m questioning the availability of the data for this manuscript. The only thing available seems to be contigs assemblies from Velvet. Where is the rest of the data used and generated in the manuscript that would be useful to the community?* *As far as I can tell the raw read information isn’t available and either is the annotations done for the assemblies. These exclusions don’t really adhere to the data availability guidelines of the journal.*

Response: We report in Genbank all the information related to this project: Bioproject, Biosample, and WGS data, which are the focus of our manuscript and put this information available to the community. In the Genbank server, we considered reporting only the best data of assemblies (contigs from Velvet) to avoid errors or confusion. Also, related to this query, in the results section, we included a sentence making explicit the availability of the assembled genome. Now we also add as supporting information the other assemblies statistics obtained in this work.

As for the raw sequence information, we are still working with that data. We appreciate your understanding of their reserve until we finish with their processing. If more information is needed for the scientific community at this stage, we ask the reviewers to convey the concern again.

*5. Why are there such differences in the assemblies? It seems reasonable for one to think that the T5S-T4 genome might have a better assembly as there is more input data, but the findings show the opposite with the T5S-T4 genome having substantially more contigs and much smaller N50. Is there an underlying data difference, characteristic of the genome or other possible reason for this?*

Response: Although the genome of *B. altitudinis* T5S-T4 has more reads, it doesn´t mean that it will generate a better assembly. The data of both genomes were obtained in the same way, and the characteristics of the reads generated were remarkably similar, so we don´t identified any underlying difference.

*6. For the T5S-T4 assembly, why was the Velvet assembly picked as the best over the ABySS assembly? Going by metrics of # of contigs it is very close and the ABySS assembly has a larger contig and a much larger N50.*

Response: About this item, we decided to select the assembly with a lower number of contigs ≥ 500 bp as better. Velvet assembler is commonly used in prokaryotic genome assembly, so we take the decision mentioned above.

*7. It seems a bit more could be done to choose which assembly is the ‘best’ than just contig number, largest contig size, and N50. Other measures that could be considered would be map the reads back to the assemblies to determine the number that align and at what mapping quality. Also, the annotation is not assessed at all, but could also be used to help determine the assembly quality by using a BUSCO or similar software to check for gene completeness. These would really help strengthen the reasoning for picking an assembly over others.*

Response: As we answered in the commentary 2, in the new version of our manuscript we include the genome quality statistics generated by different assemblers as Supplementary material.

*8. Figure 3 and 4: Is there are better way the authors can think of to present this data? The pie chart does’t really add anything and it is difficult to match up colors of categories and the chart. The authors could perhaps at least order the output and categories by size or some other manner for easier comparisons for the readers.*

Response: The information from Figures 3 and 4 were replaced and reorganized in Table 5 for better understanding.

*9. pg 4 line 88-89: What region are you referring too? The reader isn’t familiar with where you are*

Response: We clarified this sentence by adding: *Ilex paraguariensis* St. Hil., a plant that is also commonly called yerba mate, is one of the most economically important crops in the northeast of Argentina.

*10. pg 5 line 108-11: What type of insights? Could expand this to help the reader understand what is known in the genomics of PGPB to see how your work is important and fits and adds to the community*

Response: We added more information about the molecular and genetic mechanisms of plant growth promoting (PGP) activities.

*11. pg 14 line 249-250: There is no highlighting the importance of filtering in this manuscript. There are no results of assemblies without trimming to compare to assemblies with trimming, so this statement can’t really be made based on the data presented in this manuscript*

Response: A citation of a previous work of our authorship was included in the discussion section to add information related to trimming and filtering steps and their effect on the genome assembly of *B. altitudinis* 19RS3. In that publication, we compare the assemblies obtained by using raw-reads and filtered-reads as input files.

*12. pg 14 line 259-262: This sentence is confusing. It is unclear how kmers were picked and how you would use kmers that are larger than some, or most, of the read lengths. If the kmer length was larger then the read was the read removed from the analysis?*

Response: We added more information about the k-mer size selection in the materials and methods section. In general terms, the values were odd to avoid palindromes and were strictly inferior to read length.

*13. Grammar and tense usage throughout the manuscript needs to be checked.*

Response: We thank Reviewers 1 and 2 for their careful reading of the manuscript and for their constructive remarks, which were useful in improving our paper. We followed this important comment and the manuscript writing was completely checked.
